# Identification of a Steric Zipper Motif in the Amyloidogenic Core of Human Cystatin C and Its Use for the Design of Self-Assembling Peptides

**DOI:** 10.3390/ijms23105800

**Published:** 2022-05-22

**Authors:** Emilia Iłowska, Jakub Barciszewski, Mariusz Jaskólski, Augustyn Moliński, Maciej Kozak, Aneta Szymańska

**Affiliations:** 1 Department of Organic Chemistry, Faculty of Chemistry, University of Gdansk, 80-308 Gdansk, Poland; 2 Institute of Bioorganic Chemistry, Polish Academy of Sciences, 61-704 Poznan, Poland; agent007@man.poznan.pl (J.B.); mariuszj@amu.edu.pl (M.J.); 3 Department of Crystallography, Faculty of Chemistry, Adam Mickiewicz University in Poznan, 61-614 Poznan, Poland; 4 Department of Biomedical Physics, Faculty of Physics, Adam Mickiewicz University in Poznan, 61-614 Poznan, Poland; augmol@amu.edu.pl (A.M.); mkozak@amu.edu.pl (M.K.); 5 NanoBioMedical Centre, Adam Mickiewicz University in Poznan, 61-614 Poznan, Poland; 6 Department of Biomedical Chemistry, Faculty of Chemistry, University of Gdansk, 80-308 Gdansk, Poland; aneta.szymanska@ug.edu.pl

**Keywords:** human cystatin C (HCC), steric zipper, self-assembly peptides

## Abstract

Amyloid fibrils have been known for many years. Unfortunately, their fame stems from negative aspects related to amyloid diseases. Nevertheless, due to their properties, they can be used as interesting nanomaterials. Apart from their remarkable stability, amyloid fibrils may be regarded as a kind of a storage medium and as a source of active peptides. In many cases, their structure may guarantee a controlled and slow release of peptides in their active form; therefore, they can be used as a potential nanomaterial in drug delivery systems. In addition, amyloid fibrils display controllable stiffness, flexibility, and satisfactory mechanical strength. In addition, they can be modified and functionalized very easily. Understanding the structure and genesis of amyloid assemblies derived from a broad range of amyloidogenic proteins could help to better understand and use this unique material. One of the factors responsible for amyloid aggregation is the steric zipper. Here, we report the discovery of steric zipper-forming peptides in the sequence of the amyloidogenic protein, human cystatin C (HCC). The ability of short peptides derived from this fragment of HCC to form fibrillar structures with defined self-association characteristics and the factors influencing this aggregation are also presented in this paper.

## 1. Introduction

Amyloid fibrils are best known for their association with several devastating disorders, including Alzheimer’s, Parkinson’s and other neurodegenerative diseases [1,2,3]. However, proteins from living organisms are also an inestimable source of inspiration for the design of drugs, inhibitors, biomaterials, nanostructures, and other materials for medical and technological applications. Amyloids, which have historically been associated with diseases, have also been recognized as biological structures that perform vital physiological functions in host organisms, highlighting their potential as life-inspired assemblies [4,5,6]. The term amyloid refers to a protein quaternary assembly characterized by a cross-β-structure organization, in which individual β-strands are nearly perpendicular to the fibril axis and the β-sheets are tightly packed around each other. The β-strands forming the β-sheets can have either parallel or antiparallel orientation. Prediction of the architecture of that structure and investigating the self-assembly mechanism of proteins is a huge challenge, because the mechanism of amyloid assembly is still poorly understood [7]. Additionally, it is difficult to work with large macromolecules and the dynamics of their self-assembly/aggregation, and it is especially problematic to characterize the structural and biophysical properties of the oligomeric intermediates. Instead, short peptides are often more suitable for studying oligomerization and amyloid self-assembly. Eisenberg and co-workers showed that it is possible to determine the atomic structure for short peptides, which play a crucial role in the stability of amyloid fibrils [8,9,10,11]. A good strategy and workflow for the design and identification of self-assembling peptides is to start from a natural amyloid-prone polypeptide and identify the minimum peptide segment that can self-assemble without the rest of the parent molecule. In our project, this natural protein was the amyloidogenic human cystatin C (HCC).

Human cystatin C is found in all body fluids, most notably in the cerebrospinal fluid, where it functions as an inhibitor of cysteine proteases, providing conditions for their homeostasis and where it also provides protection against invasion of pathogenic microorganisms. HCC is also an amyloidogenic protein [12]. The amyloidogenic properties are dramatically increased in people with hereditary cystatin C amyloid angiopathy (HCCAA), where the L68Q mutation in the protein sequence leads to massive amyloid deposition in the brain arteries, cerebral hemorrhage, and early death [13]. The amyloidogenic properties of HCC have been conclusively linked to 3D domain swapping [14,15], first confirmed in HCC dimers by Janowski et al. [16]. The structure of monomeric HCC is known thanks to genetically engineered disulfide bonds [17], or a point mutation [18,19] in a key loop (L1) that stabilizes the monomeric fold against 3D domain swapping. The topology of the monomeric fold consists of a curved five-stranded β-sheet crossed on the concave side by a long α-helix, connected according to the (N)-β1-α-β2-L1-β3-AS-β4-L2-β5-(C) pattern, where the N-terminal peptide and loops L1 and L2 form a sequential epitope for docking in the active site of papain-like cysteine proteases. AS is a broad, poorly structured loop (appending structure) harboring a binding site for an unrelated inhibitory activity against legumain [20]. Upon 3D domain swapping, which is one of the possible mechanisms of protein oligomerization and aggregation [21,22], protein molecules exchange elements of their tertiary structure, leading to the recreation of the monomeric topology (resembling the ‘closed monomer’) from more than one polypeptide chain.

3D domain swapping is not the only proposed mechanism of amyloid fibril formation. Another mechanism involves the formation of an infinite steric zipper, first described by Eisenberg et al. [8] A classic steric zipper is formed by endless repetition of a zipper-prone sequence of approximately six residues, arranged into two identical infinite parallel β-sheets, which face each other across a 2_1_ screw, forming a tightly packed dry interface [8]. On the outside, the β-sheets are reinforced by β-sheet-like interactions of the amide groups of the Asn and Gln side chains, which are typical elements of a steric zipper sequence signature. Steric zipper formation is not necessarily an exclusive alternative incompatible with 3D domain swapping as a mechanism of amyloid fibril formation, but it has been proposed [23,24] that the two modes of aggregation could operate together. Cystatin C seems to be the case where these two phenomena meet together to boost the amyloidogenic propensity of the molecule.

**Figure 1:**
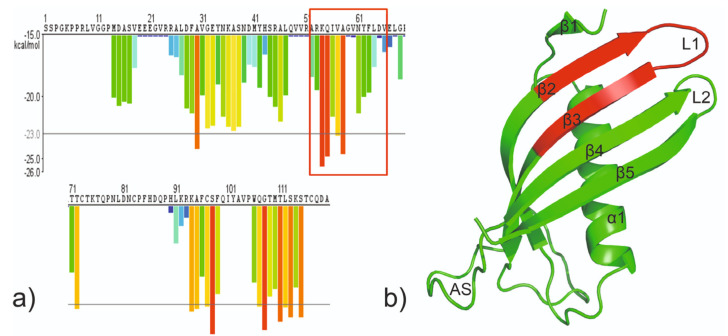
(**a**) The 3D profile prediction of fibrillizing segments in HCC sequence. The predicted lowest energy template interactions according to ROSETTADESIGN energy calculation are plotted at the initial residue of each hexapeptide. Red and orange bars represent sequences of the hexapeptides which have tendency to form fibrils. Yellow, green, and blue bars represent sequences of the hexapeptides that are predicted not to form fibrils [25]. (**b**) The crystal structure of the monomeric mutant V57N (PDB: 3XN0; [18]) with marked fragments fulfilling potential conditions of steric zipper; red: Ala^52^ −Asp^65^.

Our earlier studies showed that there is indeed a steric zipper motif region in human cystatin C spanning residues from Ala^52^ to Asn^65^ that coincide with so-called hinge loop L1, involved as a structural switch in the domain swapping (Figure 1) [25]. However, without detailed structural studies, it is difficult to determine exactly which sequence forms the steric zipper motif. Based on our studies—aggregation test [25] and crystallization trials (data not published) [26]—we selected the two most promising peptides, ^55^QIVAGV^60^ (CysZ4) and ^60^VNYFLD^65^ (CysZ9), for detailed studies aimed at the evaluation of their potential to serve as an effective steric zipper driving the self-association and fibrillization of human cystatin C. To this end, structural studies such as atomic force microscopy (AFM), crystallization trials, and X-ray fiber diffraction were used, and the results of this part of our research are presented in this publication.

## 2. Results

### 2.1. Peptide Design, Synthesis and Purification

In our previous work, we identified two consecutive steric zipper motives in the sequence of human cystatin C. They are located back to back in the region 55–65 and are comprised of six amino acid residues each. According to Eisenberg’s hypothesis, the steric zipper properties depend on a particular amino acid sequence, and its change (“scrambling”) should strongly impact the self-assembling properties [11,25,27]. Therefore, we decided to obtain two shuffled peptides with the sequences VIGAQV (CysZ11) and QAGIVV (CysZ13) and check their ability to form fibrils. All designed sequences are presented in Table 1.

### 2.2. Size Exclusion Chromatography (SEC)

**Figure 2:**
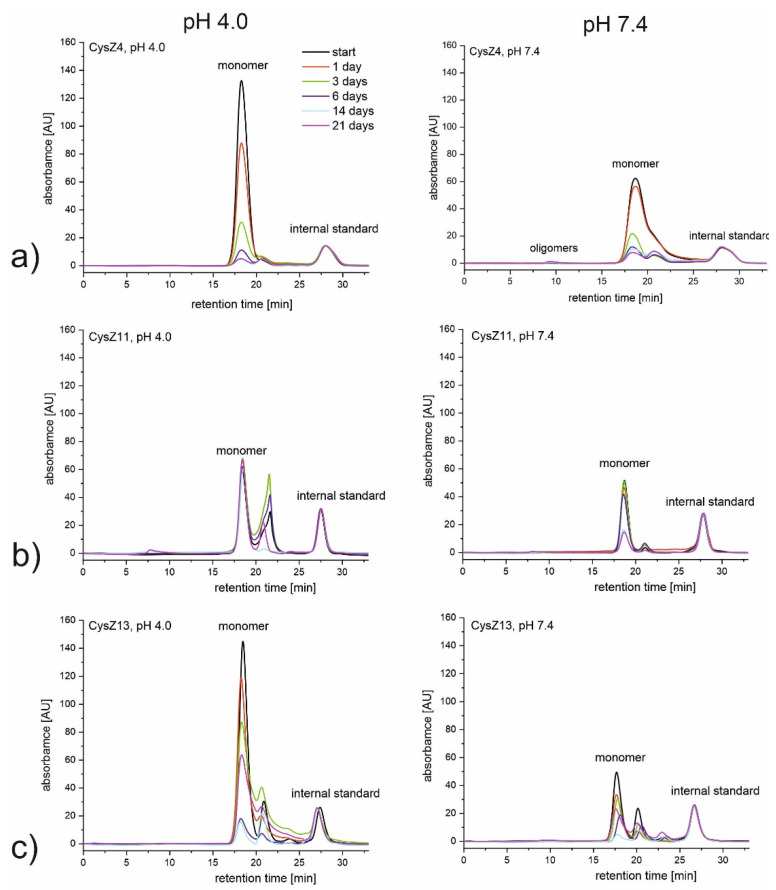
SEC chromatograms recorded during 21 days of incubation at 37 °C in PBS pH 4.0 (left side) and pH 7.4 (right side) for peptides (**a**) CysZ4; (**b**) CysZ11; and (**c**) CysZ13.

To study the changes in the soluble forms of the investigated peptides, the method of size exclusion chromatography was applied. The results of these experiments are presented in Figure 2. Peptide CysZ4 was used in all experiments as a reference (Figure 2a). All freshly dissolved peptides are in monomeric form. The retention time corresponding to the monomers varies and depends on the mass and structure of the peptide; nevertheless, it oscillates between 18 and 19 min.

In acidic conditions, only the scrambled peptide, CysZ11, was stable, while all the other peptides precipitated from the solution upon incubation (Figure 2b). Precipitate was clearly visible in the tubes, and we observed a decrease in the intensity of the signal belonging to the monomers on the SEC chromatograms. For the second scrambled peptide, CysZ13, a significant decrease in the intensity of the monomeric form up to day 14 of incubation was observed. A prolonged incubation, up to 21 days, led, however, to some kind of equilibrium between the monomeric and oligomeric forms of the peptide, since some increase in the monomeric content was noted (Figure 2c). At pH 7.4, a decrease in monomer concentration for each of the tested peptides was registered; nevertheless, CysZ11 again showed the highest stability.

### 2.3. Circular Dichroism Measurements

**Figure 3:**
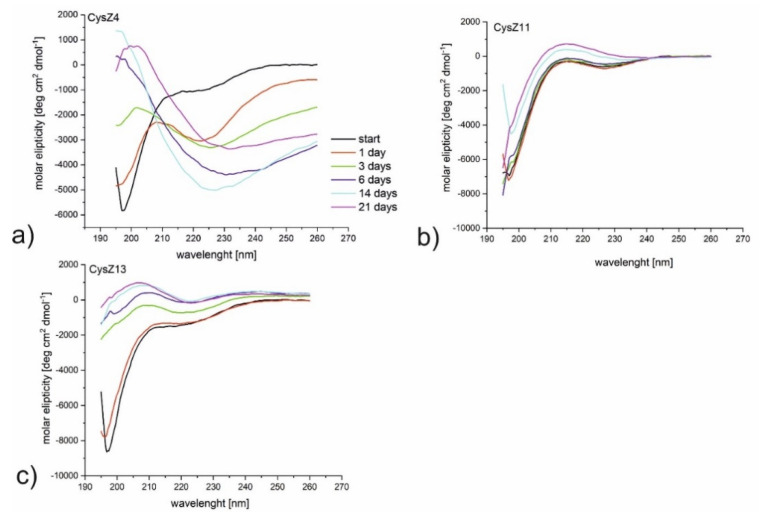
CD spectra for the peptides (**a**) CysZ4, (**b**) CysZ11, and (**c**) CysZ13 after 21 days incubation at a concentration of 1 mg/mL in PBS pH 7.4.

To understand the conformational conversions during the fibrillization pathway, we employed CD measurements to examine secondary the structure content of the studied peptides. We monitored conformational changes during 21 days of incubation at 37 °C with constant agitation. Because of the observed precipitation in the samples, before the CD measurements, all samples were centrifuged, and the CD spectra of the clear solution were then measured. The CD spectrum for CysZ4 (reference peptide) recorded freshly after dissolution indicates a random conformation with a minimum below 200 nm (Figure 3a). However, already after one day of incubation, the conformation of the peptide changes to a form for which a new local maximum at 203 nm and a broad minimum at 240 nm can be observed. Even though a CD spectrum with this shape can hardly be associated with classic β-sheet, which typically has a positive maximum at 195 nm and a negative minimum at about 216 nm [28], the conformational transition is evident. Similar conformational changes, but after three days of incubation, were observed for CysZ13 (Figure 3c). These spectra are typical for the β-sheet with a maximum between 195 and 200 nm and a minimum at about 225 nm, the presence of a minimum for CysZ13 at~220 nm is more spectacular than for the CysZ4 peptide. The last peptides from this series, CysZ11 (Figure 3b), formed a random coil conformation during the whole experiment.

### 2.4. Thioflavin T Assay

**Figure 4:**
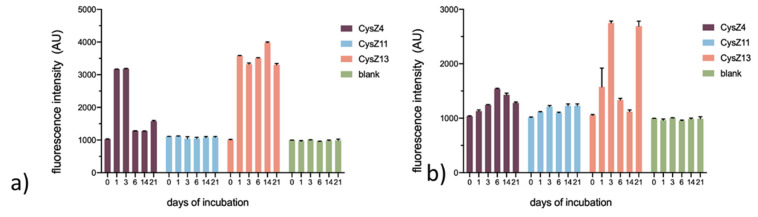
Dependence of the fluorescence intensity signal of the peptide-thioflavin T complex at 482 nm on the time of incubation relative to a control (Thioflavin T–blank) in PBS (**a**) pH 4.0; (**b**) pH 7.4. Results are presented as mean and standard error of mean (SEM).

The fibrillization process was monitored by a Thioflavin T (ThT) assay. At acidic pH, which is routinely used to induce dimerization and oligomerization of HCC [14] (Figure 4a), the fibril formation process was much faster and more efficient for CysZ4 and CysZ13 than at higher pH. We observed an increase in the fluorescence intensity signal at 482 nm already after one day, and the ThT fluorescence intensity was much higher than in neutral conditions. For the scrambled peptide, CysZ11, we did not observe fibril formation, reported by ThT emission, under any of the studied conditions. At neutral pH (Figure 4b), the process for the CysZ13 peptide was more efficient than for the other compounds. Thioflavin T and fibrillated CysZ13 formed a complex that could be detected by a significant increase in the fluorescence intensity of the dye, with an emission maximum observed at 482 nm, already after three days of incubation in PBS pH 7.4. Prolonged incubation of the sample, however, led to a significant decrease in the ThT emission intensity to values close to the background level and a re-increase in fluorescence after 21 days of this process. This phenomenon was accompanied by a strong increase in the turbidity of the sample and the formation of aggregates visible to the naked eye. Thus, the disappearance of the ThT signal can be explained by the massive precipitation of the peptide. Additionally, peptide CysZ4 showed an increase in ThT fluorescence intensity, but to a lesser extent than CysZ13. The maximum response was achieved after six days of incubation. The process of fibril formation was slower, and precipitation was also observed in the tubes.

### 2.5. Transmission Electron Microscopy

**Figure 5:**
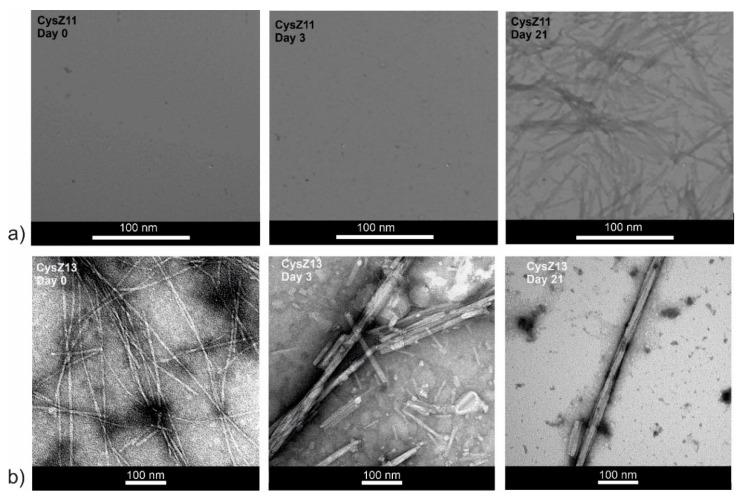
Transmission electron micrographs (TEM) for peptides (**a**) CysZ11 and (**b**) CysZ13, taken before, on the 3rd day, and after 21 days of incubation in PBS buffer pH 4.0.

**Figure 6:**
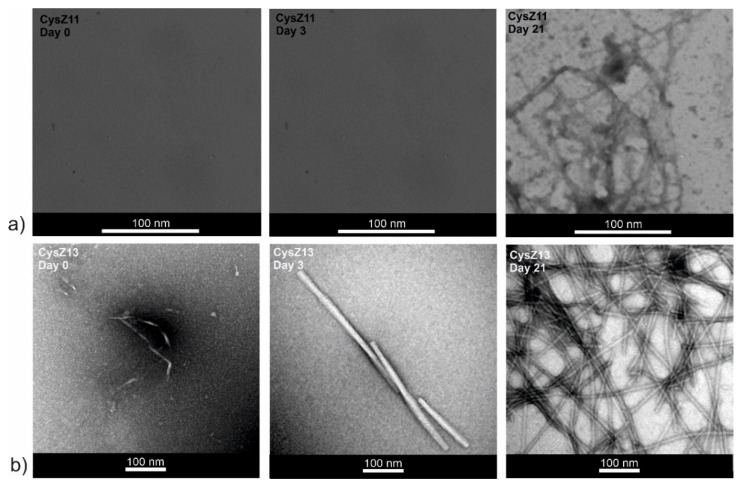
Transmission electron micrographs (TEM) for peptides (**a**) CysZ11 and (**b**) CysZ13, taken before, on the 3rd day, and after 21 days of incubation in PBS buffer pH 7.4.

To confirm the formation of amyloid fibrils for the investigated peptides and to characterize other possible oligomeric assemblies present in the samples, a TEM analysis was carried out. Figure 5 and Figure 6 present the micrographs for CysZ11 and CysZ13 peptides. It can be seen that CysZ11, during the first day of incubation under both studied conditions, did not form any fibrils. Small protofilaments with a diameter of ~2 nm were observed after 21 days in both experiments (Figure 5a and Figure 6a). This result confirms the low propensity of this shuffled analogue to fibril formation. The second scrambled analogue, CysZ13 fibrillized more easily than the reference peptide CysZ4, regardless of the conditions of the experiment. In acidic buffer (PBS pH 4.0), many twisted protofilaments with diameter ca. 18 nm were already present in the solution at the beginning of the experiment. After 3 days of incubation, tubular structures with a diameter of 24 nm that looked like microcrystals were visible. Finally, after 21 days of the process, regular microcrystals and ribbon-like twisted fibrils could be observed (Figure 5b).

At pH 7.4, the opposite situation was encountered. After dissolution, some protofilaments with a diameter of ~10 nm were observed. Prolonged incubation led to a change of the fibril morphology, first into rod-like structures resembling microcrystals with a diameter of ~24 nm (after 3 days). Finally, after 21 days of the process, more fibrils with a diameter of ~18 nm (Figure 6b) were formed.

### 2.6. Atomic Force Microscopy

**Figure 7:**
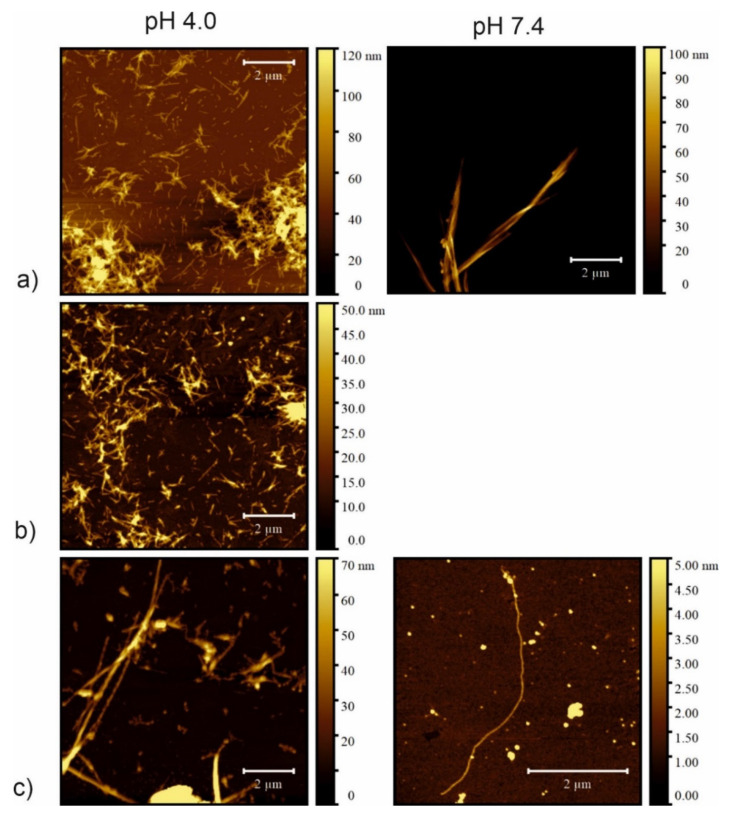
Atomic force microscopy (AFM) images of fibrils taken after 21 days of incubation in PBS buffer: left column pH 4.0; right column pH 7.4 of the peptides (**a**) CysZ4; (**b**) CysZ9 and (**c**) CysZ13.

The second technique used to visualize and compare the morphology of the fibrils was atomic force microscopy (AFM). AFM imaging is a complementary method to TEM, because it allows a more precise assessment of the height of the obtained fibrils. The AFM images of raw incubation mixtures also showed that dense, three-dimensional networks of peptide fibrils were formed that completely cover the mica surfaces (Supplementary Materials, Figure S1), despite the small amount of sample applied. The AFM images were recorded for samples after 21 days of incubation and after four water washings. This procedure was conducted to wash off the excess salt present in the samples and to dilute the samples so that there was a chance of examining the structure of the peptide fibrils more precisely. The images thus obtained showed a variety of fibrillar structures. Concerning sample CysZ4 (Figure 7a), there is a clear difference in the structure and behavior of the fibrils formed under different conditions. At pH 4.0, a great number of fibrils varying in both diameter (from 15 to 60 nm) and length were observed. Moreover, some of these fibrils were clearly composed of smaller ones twisted in a symmetric manner. In contrast, at pH 7.4, CysZ4 formed much longer fibrils with a diameter similar to those formed at pH 4.0, but which tended to aggregate into structures as high as 100 nm, still keeping the tendency to twist around each other. For sample CysZ9 (Figure 7b), fibrils were not observed at pH 7.4, whereas at pH 4.0 the fibrils were similar in both structure and behavior to those formed by CysZ4. For CysZ11, fibrils were observed at neither pH 4.0 nor 7.4. Finally, sample CysZ13 (Figure 7c) showed the biggest dissimilarity, depending on pH of the solution. At pH 4.0, it formed long fibrils with diameter varying from 15 to 90 nm, aligned in a twisted manner, whereas at pH 7.4 we were able to observe just one long fibril, not aggregated in any way, with a diameter of 2 nm.

### 2.7. Attempts to Determine the Crystallographic Structure

Some of the peptides studied by us previously had the tendency to form microcrystal-like assemblies [25]. Therefore, we decided to check whether they can be crystallized. For this purpose, we selected peptides CysZ4 and CysZ9 for crystallization trials. The experiments were set up in hanging drop vapor diffusion mode and included exploring commercial screen conditions. Unfortunately, the most interesting peptide, CysZ4, precipitated in all experimental conditions, and we were unable to obtain it in crystalline form. We noted that the precipitated CysZ4 peptide, under polarized light, exhibited the so-called Maltese cross effect, which is typical of self-assembling fibrils [29,30] (Supplementary Materials, Figure S2).

**Figure 8:**
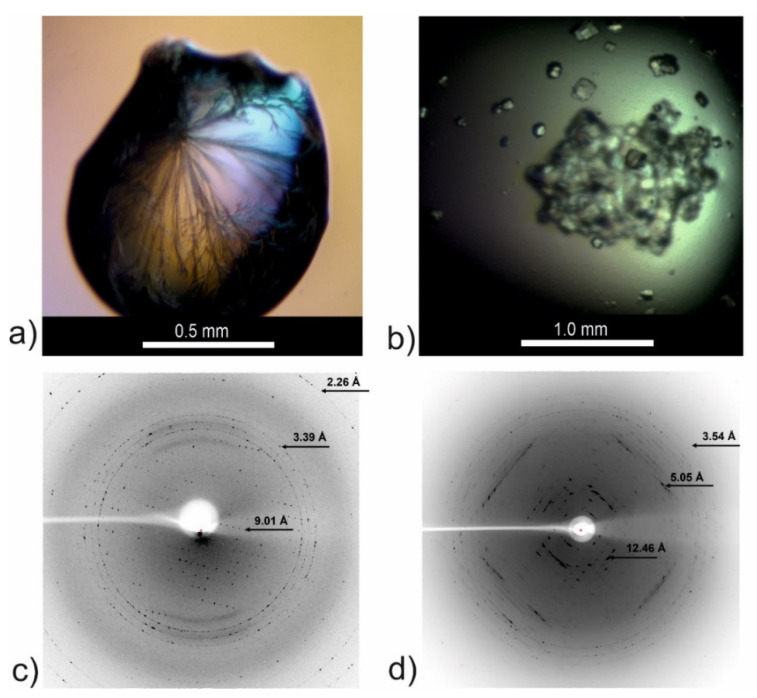
Crystallization drops and X-ray diffraction images for the CysZ9 peptide crystallized in (**a**) needle-shaped form from 2.4 M sodium malonate pH 6.0, and in (**b**) cuboid form from 0.2 M MgCl_2_, 10% MPD, 0.05 M sodium cacodylate pH 6.5, 0.001 M spermine. (**c**) Sample diffraction image recorded for crystal from (**a**); (**d**) sample diffraction image recorded for crystal form (**b**).

Microcrystals were obtained for the CysZ9 peptide, which corresponds to the “second half” of our tandem steric zipper [25]. Details of the crystallization conditions and of the attempts to determine the crystal structure can be found in Figure 8 and in the methods section. The crystals grew as either thin needles (Figure 8a), cuboids (Figure 8b), or, most often, as conglomerates of plates. For the needle and cuboid crystals, we collected X-ray diffraction data using the BESSY II synchrotron in Berlin. As can be seen from Figure 8c,d, the highest resolution recorded was 2.26 Å for the needles (Figure 8c) and 3.54 Å for the cuboids (Figure 8d), respectively. Unfortunately, the diffraction data were of poor quality and incomplete, making the full crystal structure determination impossible. Using the HKL3000 package [31,32] we were able to process data for cuboid crystals, which allowed us to determine the space group as monoclinic *C*2 and the unit cell parameters (Table 2). The calculated volume of the unit cell was ~218,800 Å^3^, suggesting the presence of numerous copies of the peptide in the asymmetric unit. For example, at the Matthews volume [33,34] of ~2.5 Å^3^/Da (corresponding to ~50% solvent content), there would be 28 copies of the peptide in the asymmetric unit. This strongly suggests that the CysZ9 peptide crystallized in an associated form, possibly as amyloid-like fibrils.

### 2.8. X-Ray Fiber Diffraction

**Figure 9:**
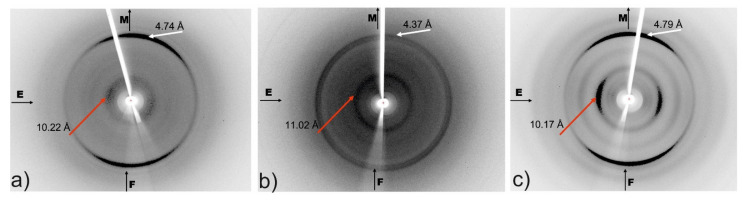
Cross-β signature in the X-ray fiber diffraction patterns. The diffraction patterns were obtained for: (**a**) CysZ4; (**b**) CysZ9 and (**c**) CysZ13 fibers; the position of the strong meridional (M) reflection is highlighted by white arrows, while the position of the strong equatorial (E) reflection is highlighted by red arrows. F–fiber axis.

X-ray fiber diffraction experiments were performed on fibers comprised of amyloid-like fibrils, derived by self-assembly of the aggregation-prone peptides CysZ4, CysZ9 and for the shuffled peptide CysZ13. The X-ray diffraction patterns indicate that the fibers formed in each case display the typical ‘cross-β’-like signature and, therefore, must possess the corresponding molecular architecture of the amyloid fibrils (Figure 9). The strong meridional reflections corresponding to a structural repeat of 4.37–4.79 Å indicate the spacing between the hydrogen bonded β-strands in the direction of the long axis of the fibril. Moreover, all the diffraction patterns exhibit a second strong reflection, located along the equator, corresponding to a d-spacing of 10.17–11.02 Å. The differences observed in the position of the equatorial reflections indicate dissimilarities in the packing distance of the peptide sheets in each case, which could be caused by the different sizes of the side chains of the amino acid residues. Peptides CysZ4 and CysZ13 are built from the same amino acid residues, only in a different arrangement, and both of them have much stronger reflections on the X-ray diffraction photographs (Figure 9a,c) than CysZ9 (Figure 9b). The equatorial reflections for the CysZ4 and CysZ13 peptides (10.11 Å, 10.17 Å) indicate a much closer packing of the β-sheets than in CysZ9 (11.02 Å). The presence of these two characteristic reflections clearly indicates the existence of β-sheets in the cross-β structure, as observed in many amyloid-like fibrils, characteristic for peptides forming the steric zipper motif [35].

## 3. Discussion

The aim of this project was to study in more detail the potential steric zipper forming peptides in the sequence of human cystatin C, and to further characterize their self-assembly propensities. Our earlier bioinformatic analyses aimed at the identification of potential steric zipper segments using the algorithm elaborated by the Eisenberg group (3D profile method), followed by experimental aggregation tests, showed that the region in the middle of human cystatin C around the flexible loop L1 has a very high potential to form the steric zipper motif [25]. The crucial role of the L1 hinge loop region in HCC oligomerization has been confirmed by numerous studies, both of the wild-type protein and its various mutants [36,37,38,39,40]. Additionally, Tsiolaki and co-workers confirmed that the region ^56^IVAGVNYFLD^65^ in the HCC sequence plays a crucial structural role in HCC fibrillation [41]. They showed that the fragment responsible for interaction in tetramers that promotes their interaction and the formation of octamers (PDB ID: 1R4C) is the pentapeptide ^47^LQVVR^51^ [42,43]. In their report, it is an ‘aggregation-prone’ segment based on AMYLPREP prediction, and the amyloidogenic properties of this peptides were confirmed. Our previous research shows that in the sequence of HCC there is at least one segment that might be responsible for its self-assembly properties. We identified two consecutive steric zipper motives located in loop L1 and the surrounding β-strands [25]. In the present work, we sought to characterize more deeply these steric zipper motives, performing more detailed structural studies, with the application of AFM and X-ray diffraction techniques.

AFM is more accurate than the previously used TEM. It showed that CysZ4 in both fibrillization conditions does indeed form twisted fibrils resembling ribbons (Figure 7a). In acidic pH, the fibrils were much smaller and shorter (ca. 1–2 μm), while at pH 7.4 they reached up to 7 µm. The twists of the fibrils are clearly visible on the images, which suggests that they must be formed from several β-sheets wrapped around each other. The use of AFM imaging also allowed us to verify that CysZ9 (Figure 7b) forms short, twisted, and interconnected peptide fibrils.

Next, we used X-ray diffraction methods to glean insight into the molecular architecture of our selected peptides. Determination of 3D atomic structure is the ultimate and irrefutable evidence for the existence of a steric zipper motif. Unfortunately, most of our crystallization trials led to precipitation of the peptides in the crystallization drops due to their very high propensity for self-association even at low concentration. For CysZ9, the crystallization trials yielded microcrystals with several morphologies, including needle-shaped whiskers. This morphology is typical and characteristic for small molecules, such as short peptides [23,24]. Under the polarizing microscope, these needles showed distinctive birefringence (Maltese cross effect, Supplementary Materials, Figure S2) [29,30]. In addition to those typical peptide microcrystals, we were also able to obtain regular crystals for which we could collect X-ray diffraction data. This indicates that the asymmetric unit could be occupied by a mature fibril comprised of many copies of the amyloidogenic peptide. The factors responsible for the stabilization of the fibril structure might be speculated to be the presence of aromatic residues, Tyr and Phe, in the sequence of CysZ9 and the aromatic interactions between them. Serpell et al. suggested aromatic interactions (π-stacking) in short peptides as crucial in amyloidogenesis [44,45]. Unfortunately, the poor resolution, quality and completeness of our diffraction data did not allow us to determine the crystal structure of the peptides, but only to characterize the symmetry and dimensions of the unit cell. It is large enough to suggest the presence of mature fibrils in the crystal. Importantly, also on the diffraction images of the single crystals for both peptides, CysZ4 and CysZ9, there are reflections at positions 4.1–5.1 Å and at 9.7–10.5 Å (Supplementary Materials, Figure S3), which is characteristic of fibrils with a cross-β structure, similar to the X-ray images reported for short fragments of, for example, Aβ or Tau proteins [46,47]. These characteristic meridional and equatorial reflections for both peptides indicate the distance between the H-bonded β-strands in the direction of the fibril and the spacing between the β-sheets in the fibril cross-section. The only difference is observed in the equatorial distance. These differences in β-sheet spacing may be due to the different amino acid sequences of CysZ4 and CysZ9. The latter peptide contains two residues with large aromatic rings (tyrosine and phenylalanine). In addition, CysZ9 contains two polar moieties, aspartic acid and asparagine, while CysZ4 is made up almost entirely of hydrophobic residues, except for one glutamine moiety. The aromatic residues could be additional factors responsible for the stabilization of the fibril structure of CysZ9.

Our work clearly showed the high fibrillization tendency of the CysZ4 fragment of human cystatin C. The fibrillization propensity of the second fragment, CysZ9, is lower than for CysZ4 and, interestingly, the buffer pH has opposite effect on it. At lower pH, which is necessary to induce effective dimerization of the full-length protein, a higher tendency for fibrillization of CysZ9 peptide was observed than at neutral pH. This may be related to the pI value of CysZ9, which is 3.9 [48].

Since the amino acid sequence plays an important role [11] in the case of the steric zipper motif, we also decided to further verify our hypothesis by generating two scrambled peptides with randomly mixed order of the amino acid residues of one of the steric zipper peptides, namely CysZ4. We selected this fragment because of its high amyloidogenic potential.

The scrambled peptides turned out to be very interesting case, because one of them (CysZ11) did indeed lose its self-assembly propensity as a result of sequence permutation, whereas the other one, CysZ13, seemed to become even more amyloidogenic. CysZ13 had significantly stronger fibrillization properties than the reference peptide, CysZ4. The SEC chromatograms recorded for samples incubated under both acidic and neutral conditions showed a clear drop in the intensity of the monomer-associated peaks up until 14 days of incubation, which was followed by an increase upon prolonged incubation, indicating that the equilibrium state of the whole fibrillization process for this peptide had been established. This was also confirmed by ThT tests, where we observed an increase followed by a decrease in ThT fluorescence intensity. However, the intensity of these signals, especially at pH 7.4, was significantly higher than for the other compounds. After only 3 days of incubation, CysZ13 changed its conformation from disordered to β-sheet, similar to that of CysZ4, as confirmed by CD spectra.

Another characteristic property of peptides with the steric zipper motif that was also observed for CysZ13 is the formation of fibrils in the form of microcrystals. We had already observed this phenomenon in TEM images for the CysZ4 peptide. In the case of the CysZ13 fibrils, the twists can be seen very well in the TEM micrographs. The optimal fibrillization conditions for this peptide were 6 days, although already after 3 days we obtained fibrils with a diameter of 11 nm and a length from 44 to as much as 400 nm. Nevertheless, the number of fibrils was not large, which is important from the point of view of their potential functionalization. However, with longer incubation of up to 21 days, the tangle of the fibrils becomes so massive that it leads primarily to their uncontrolled precipitation from the solution. The AFM images also showed that the compound did indeed have a high tendency to fibrillize. With a sufficient number of washes, it was even possible to separate a single fibril. The recorded fiber diffraction images provided further evidence that the designed peptides form amyloid fibrils. The presence of the characteristic meridional and equatorial reflections and the absence of buffer rings are consistent with a classic diffraction pattern of an amyloid peptide fibril [1,45,49,50,51,52].

Their simple molecular architecture favors the use of amyloid folds as a building block for the de novo design of polypeptide-based nanomaterials. In addition, the nanomechanical properties of amyloid structures are also important for nanomaterials design. Experimental and in silico studies have suggested that individual fibrils, owing to their dense backbone H-bond network, possess high stiffness with an elastic modulus in the GPa range [53]. Among many advances in the design of supramolecular structures [54], amyloid-based nanofibers have also been reported [55,56,57]. These are a successful example of the “bright side of the amyloid fibrils”. They are a molecular, stable sheets which can be used as self-assembly drug delivery system. In view of such recent trends in protein and peptide engineering, a comprehensive understanding of the detailed properties of amyloid assembly is expected to contribute not only to the prevention and treatment of amyloid-related diseases, but also to the exploration of amyloid functionality, with the ultimate goal of developing new nanomaterials for biological functions.

## 4. Materials and Methods

### 4.1. Peptide Synthesis

Fmoc amino acids were purchased from Peptides International (Louisville, KY, USA) and Chem-Impex (Wood Dale, IL, USA). The resin for peptide synthesis, TentaGel R RAM was purchased from Rapp Polymere GmbH (Germany). All solvents used (N,N-dimethylformamide (DMF), dichloromethane (DCM), N-methylpyrrolidone, Triton X-100, methanol, diisopropylamine, piperidine, diethyl ether, trifluoroacetic acid (TFA)), reagents (2-(1H-Benzotriazole-1-yl)-1,1,3,3-tetramethylaminium tetrafluoroborate (TBTU), N,N-diisopropylamine (DIPEA)) were obtained from Sigma-Aldrich (Saint Louis, MO, USA).

Peptides were synthesized by the standard solid-phase method in a manual microwave reactor Plazmatronica RM 800 (Plazmatronika, Poland), using N-Fmoc protected amino acids. Fmoc Rink Amide (LL) TentaGel R RAM resin was used with capacity 0.18 mmol/g. For the synthesis by Fmoc methodology, standard reagents were used, such as 20% piperidine solution in DMF for the deprotection. To form the peptide bonds, a mixture of TBTU (3.8 eq) and DIEPA (2 eq) solution in DMF was used. Peptides were purified by preparative high-performance liquid chromatography on a reversed-phase (RP-HPLC) column (Luna C18, 5 µm, 100 Å, 20 × 250 mm, Phenomenex, Torrance, CA, USA) and analyzed using analytical RP-HPLC (Jupiter^®^ C18 column, 5 μm, 300 Å, 4.6 × 250 mm, Phenomenex, Torrance, CA, USA), and mass spectrometry (ESI LCMS IT TOF, Shimadzu, Jupiter^®^ C18 column, 5 μm, 300 Å, 4.6 × 250 mm, Phenomenex, Torrance, CA, USA). A linear gradient of 80% acetonitrile (B) was applied as a mobile phase.

### 4.2. Fibril Preparation

All peptides were dissolved in Eppendorf (Hamburg, Germany) Low Retention Tubes in 1.0 mL of PBS pH 7.4 and pH 4.0. The concentration of the peptide solutions was 1 mg/mL. The peptides were incubated at 37 °C with constant orbital shaking. Aliquots for the evaluation of the fibrillization process were taken before incubation and after 1, 2, 3, 6, 14, and 21 days.

### 4.3. Size Exclusion Chromatography

Peptides were dissolved in 1 mL of PBS at pH 7.4 or 4.0 containing 0.01 mg/mL benzamidine hydrochloride (Sigma-Aldrich, Saint Louis, MO, USA) as internal standard. The concentration of all peptides was 1.5 mg/mL except CysZ9, for which it was 3 mg/mL. Peptides were incubated at 37 °C with constant agitation for 21 days. To check the progress of fibrillization, samples were centrifuged at 14,000 rpm for 5 min and 10 µL of the supernatants were injected into Superdex Peptide PC 3.2/30 column (GE Healthcare, Chicago, IL, USA) run in PBS pH 7.4 buffer at a flow rate of 0.1 mL/min. The elution of compounds was monitored spectroscopically by absorbance measurements at 223 nm. Incubation mixtures were probed after 0, 1, 3, 6 14 and 21 days.

### 4.4. Circular Dichroism

CD spectra were recorded on a Jasco J-815 spectropolarimeter (Jasco, Victoria, BC, Canada). Far-UV spectra (195–250 nm) were recorded for all peptides dissolved in PBS pH 7.4 at a concentration of 1 mg/mL and incubated at 37 °C with agitation. Spectra were recorded before and after 1,2, 3, 7, 10 and 14 days of incubation. All spectra were corrected for buffer signal. The CD spectra were recorded in triplicate, using a 0.02 mm cell. All measurements were originally in millidegrees and were converted to units of mean residue ellipticity (MRE).

### 4.5. Thioflavin T Fluorescence Assay

Ten microliters of peptide solution incubated under fibrillization conditions (see Fibril preparation) was mixed with 10 µL of 1.5 mM ThT (Sigma Aldrich, Saint Louis, MO, USA) in water and 90 µL of PBS pH 7.4 or pH 4.0. The fluorescence was measured on the Infinite 200Pro (TECAN, Group Ltd., Männedorf, Switzerland) spectrofluorometer in 96-well plates (Costar Black^®^), with excitation wavelength set at 420 nm and emission in the range of 455 to 600 nm.

### 4.6. Transmission Electron Microscopy

Peptide samples incubated under fibrillization conditions (see Fibril preparation) (5 µL) were applied on glow-discharged carbon-coated copper grids (400 mesh). After 1 min of adsorption, excess liquid was removed by blotting, and the samples were stained with 2% (v/v) aqueous uranyl acetate. The samples were examined with a TECNAI SPIRIT BIO TWIN FEI (FEI Company, Hillsboro, OR, USA) at 120 kV, with nominal magnification between 11,500 and 39,000. The assay was performed for samples before (after dissolution) and after 3 days of incubation.

### 4.7. Atomic Force Microscopy

Peptide samples were incubated under fibrillization conditions (see Fibril preparation). Next, 50 µL of the fibrillized peptide sample was diluted with 100 μL of the MiliQ water and centrifuged for 10 min (14,000 rpm, Microfuge 16, Beckman Coulter, Brea, CA, USA). Next, 80 μL of the supernatant was removed and the procedure was repeated four times. A small drop of the remaining solution was then applied to a freshly cleaved mica, rinsed after 30 s with 100 μL of MIliQ water and left to dry in air. The topography of deposited samples was analyzed using a NanoWizard^®^ 4 (JPK, Berlin, Germany) atomic force microscope. The measurements were performed in QI Mode (Quantitative Imaging Mode) using Tap150AL AFM cantilevers (Ted Pella, Inc., Redding, CA, USA). The AFM images were processed and analyzed using the Gwiddion 2.60 program package [58].

### 4.8. Crystallization and X-ray Diffraction

The crystallization trials were carried out for two peptides, CysZ4 and CysZ9. Lyophilized samples were dissolved in MiliQ water to the concentration of 2 mg/mL (CysZ4) or 4 mg/mL (CysZ9). Samples were centrifuged for 10 min (14,000 rpm, Microfuge 16, Beckman Coulter, Brea, CA, USA). The precipitate was discarded, and the supernatant was used for the crystallization trials by the hanging-drop vapor-diffusion method at 18 °C. CysZ9 was crystalized in two conditions.

The first was 2.4 M sodium malonate pH 6.0. A single needle-shaped whisker crystal was cryoprotected in mother liquor supplemented with 70% (*v/v*) MPD and then flash-vitrified at 100 K in nitrogen gas stream.

The second was 0.2 M MgCl_2_, 10% MPD, 0.05 M sodium cacodylate pH 6.5, 0.001 M spermine. A cuboid single crystal with the dimensions of 0.13 mm was cryoprotected in mother liquor supplemented with 60% (*v/v*) MPD and then flash-vitrified at 100 K in nitrogen gas stream. For both crystals, X-ray diffraction data were collected using synchrotron radiation at beamline 14.2 of the BESSY II synchrotron (Berlin, Germany), equipped with a Rayonix MX-225 square CCD detector. The diffraction data were processed using HKL3000 [31,32].

### 4.9. X-ray Fiber Diffraction

Fibrils were formed by incubating the peptides in PBS pH 7.4 (CysZ4 and CysZ13) or pH 4.0 (CysZ9) at a concentration of 10 mg/mL for 21 days at 37 °C with agitation. Fibrils of CysZ4 and CysZ9 were sedimented by centrifugation at 14,000 rpm for 20 min (Microfuge 16, Beckman Coulter, Brea, CA, USA) to remove supernatant. A small part of the precipitate was placed in a crystallographic cryo-loop with 0.2 mm diameter. The sample-detector distance was 180 mm and the exposure time was 30 s. The φ step was 8°. The fibril microcrystals were obtained after the 21-day incubation process. After a sample was centrifugated, most of supernatant was removed, and a single fibril microcrystal of CysZ13 was fished out from the suspension using a crystallographic cryo-loop with 0.2 mm diameter. The experimental setup was as follows: detector distance 250 mm, exposure time 8 s, φ step 8 °. All X-ray fiber diffraction data were collected at beamline 14.3 of the BESSY II synchrotron in Berlin at 20 °C, using 0.894 Å wavelength. The X-ray patterns were displayed and analyzed using the ADXV (ADXV (Area Detector Systems Corporation; Poway, CA, USA) [59].

## Figures and Tables

**Table 1:** Amino acid sequences of the peptides.

Peptide ID	Kind of the Sequence	Amino Acid Sequence
**CysZ4**	HCC 55–60	NH_2_-QIVAGV-NH_2_
**CysZ9**	HCC 60–65	NH_2_-VNYFLD-NH_2_
**CysZ11**	shuffle HCC 55–60	NH_2_-VIGAQV-NH_2_
**CysZ13**	shuffle HCC 55–60	NH_2_-QAGIVV-NH_2_

**Table 2:** Crystallographic data and data collection parameters for CysZ9.

Synchrotron/Beamline	BESSY II/14.2
Temperature (K)	100 K
Space group	*C*2
Cell parameters (Å,°)	a = 71.56; b = 65.15; c = 48.01, β = 102.20
Wavelength (Å)	0.91841
Resolution (Å)	50.0−2.94 (2.99−2.94) ^a^
Reflection collected	9550
Unique reflections	4775
R_merge_	0.233 (0.646)
<I>/<σI>	3.7 (1.2)
Completeness (%)	49.6 (43.7)
Redundancy	2.0 (1.7)

^a^ Values in parentheses correspond to the highest resolution shell.

## Data Availability

The datasets supporting the conclusions and description of a complete protocols can be found within the manuscript and its additional files. The datasets used and/or analyzed during the current study are available on request from the corresponding author.

## Supplementary Materials

The following supporting information can be downloaded at: https://www.mdpi.com/article/10.3390/ijms23105800/s1.
