# Supplementary material for: Identification of a Steric Zipper Motif in the Amyloidogenic Core of Human Cystatin C and Its Use for the Design of Self-Assembling Peptides
Source: Int J Mol Sci. 2022 May 22;23(10):5800. doi: 10.3390/ijms23105800 (PMC9147961; doi:10.3390/ijms23105800)
Supplement: Supplementary file 1 [file ijms-23-05800-s001.zip › ijms-1728925-supplementary.pdf]

International Journal of *Molecular Sciences*

MDPI

*Supplementary materials*

*Article*

# Identification of a Steric Zipper Motif in the Amyloidogenic Core of Human Cystatin C and Its Use for the Design of Self-Assembling Peptides

**Emilia Iłowska [1,*], Jakub Barciszewski [2], Mariusz Jaskólski [2,3], Augustyn Moliński [4,5], Maciej Kozak [4] and Aneta Szymańska [6]**

1 Department of Organic Chemistry, Faculty of Chemistry, University of Gdansk, 80-308 Gdansk, Poland
2 Institute of Bioorganic Chemistry, Polish Academy of Sciences, 61-704 Poznan, Poland; agent007@man.poznan.pl (J.B.); mariuszj@amu.edu.pl (M.J.)
3 Department of Crystallography, Faculty of Chemistry, Adam Mickiewicz University in Poznan,
61-614 Poznan, Poland
4 Department of Biomedical Physics, Faculty of Physics, Adam Mickiewicz University in Poznan,
61-614 Poznan, Poland; augmol@amu.edu.pl (A.M.); mkozak@amu.edu.pl (M.K.)
5 NanoBioMedical Centre, Adam Mickiewicz University in Poznan, 61-614 Poznan, Poland
6 Department of Biomedical Chemistry, Faculty of Chemistry, University of Gdansk, 80-308 Gdansk, Poland;
aneta.szymanska@ug.edu.pl
* Correspondence: emilia.ilowska@ug.edu.pl

International Journal of
*Molecular Sciences*

MDPI

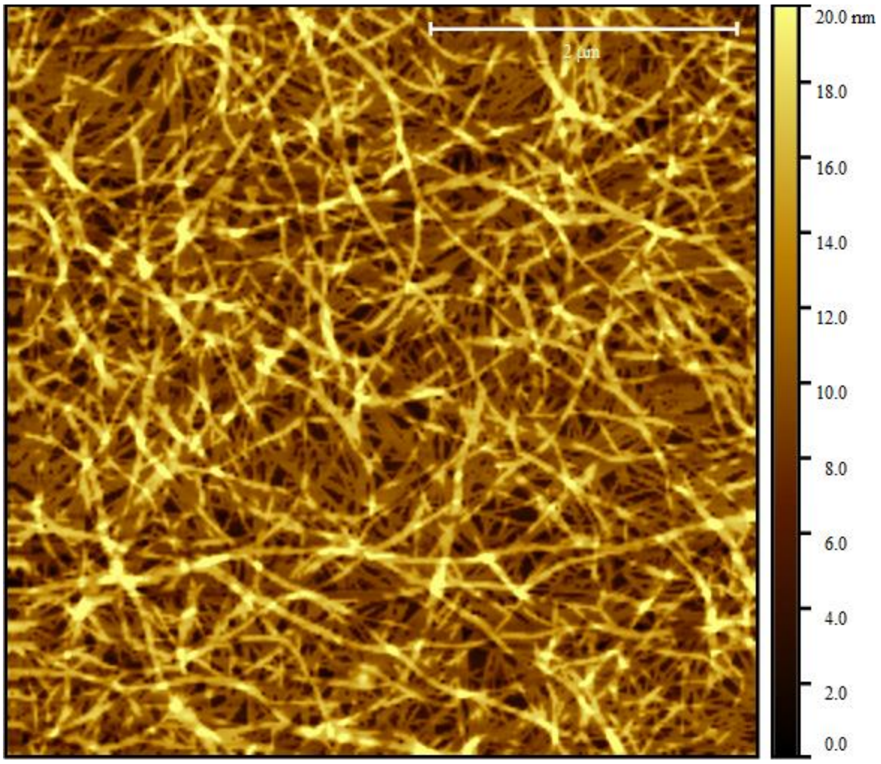


**Figure. S1**. AFM image of CysZ13 after 21 days of incubation in PBS pH 7.4, concentration 1 mg/mL, 37 °C with agitation without washing before AFM analysis (see Atomic Force Microscopy, at section Materials and Methods).

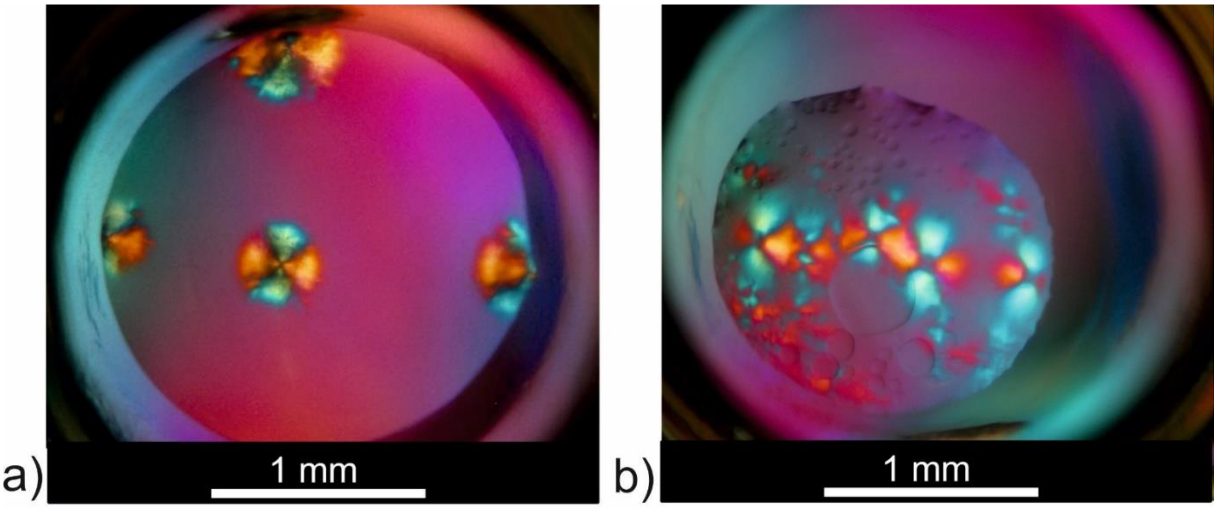


**Figure S2:** The crystallization drop of peptide (a) CysZ4 and (b) CysZ9. Under most crystallization conditions, precipitation and the "Maltese cross effect" were observed. Crystallization conditions: CysZ4: 1.9 M sodium malonate pH 6.0, C = 4 mg/mL; CysZ9: 2.4 M sodium malonate pH 5.0, C = 6 mg/mL.

International Journal of
*Molecular Sciences*

MDPI

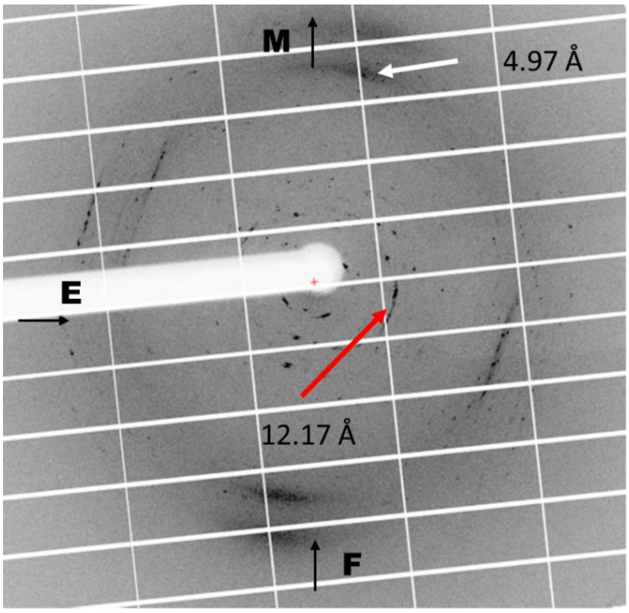


**Figure S3:** A diffraction image recorded for a crystal of CysZ9 with the strong meridional and equatorial reflections highlighted by white and red arrows, respectively. Crystallization conditions: 0.1 M $MgCl_2$, 100 mM sodium cacodylate, 15 % MPD, C = 4 mg/mL. A single (cuboid) crystal was cryoprotected in mother liquor supplemented with 60 % (v/v) MPD and flash-vitrified at 100 K in a nitrogen gas stream. X-Ray diffraction data were collected using synchrotron radiation at beamline 14.1 of the BESSY II synchrotron, equipped with Dectris Pilatus 6M.
